# Supplementary material for: Different temporal trends in vascular plant and bryophyte communities along elevational gradients over four decades
Source: Ecol Evol. 2022 Aug 22;12(8):e9102. doi: 10.1002/ece3.9102 (PMC9395318; doi:10.1002/ece3.9102)
Supplement: Supplementary file 4 — Appendix S1 Supporting Information [file ECE3-12-e9102-s003.docx]

**Supplementary material S1 - Climatic temporal trends**

To model the mean annual temperature trend in both parks, we extracted temperature data from the ANUSPLINE model (McKenney et al. 2011). The relevant period to study the effect of temperature change on forest plant communities includes a lag of approximately ~10 years before the time of the survey. We thus considered the period 1960-2005, which aligns with a previous study showing a sharp warming gradient in the region (Yagouti et al. 2008). The model includes the effect of the categorical variable park (with two levels: Forillon and Mégantic), the continuous variable year from 1960 to 2005, and a random effect on the different measures within each park.

We only report results for annual mean temperature but found similar results for annual minimum and maximum temperatures, with no temporal change in annual mean precipitation. For the period 1960-2005, we find that Forillon experienced an increase of 0.12 ^o^C ± 0.010^o^C/decade, while at Mégantic the increase per decade was almost twice as strong: 0.20 ^o^C ± 0.014^o^C/decade.

|  | **Mean annual temperature** | | | |
| --- | --- | --- | --- | --- |
|  | *Estimates* | *Sdt.Error* | t | *p* |
| Intercept (Forillon) | -29.04 | 7.505 | -3.87 | **<0.001** |
| Year | 0.012 | 0.001 | 11.88 | **<0.001** |
| Year : Mégantic | 0.008 | 0.001 | 5.49 | **<0.001** |
| **Random Effects** |  |  |  |  |
| Variance residual | 0.436 |  |  |  |
| Variance  _plot:park_ | 0.429 |  |  |  |
| Variance  _park_ | 108.725 |  |  |  |
| Marginal R^2^ / Conditional R^2^ | 0.340 / 0.997 |  |  |  |
